# Phase IIB Randomized Trial on the Use of 4-Aminopyridine in Guillain-Barré Syndrome

**DOI:** 10.1016/j.arrct.2021.100123

**Published:** 2021-04-08

**Authors:** Jay M. Meythaler, Robert C. Brunner, Jean Peduzzi

**Affiliations:** a Department of Physical Medicine and Rehabilitation-Oakwood, Wayne State University School of Medicine, Vestavia Hills, Alabama; b Department of Physical Medicine and Rehabilitation, University of Alabama at Birmingham School of Medicine, Birmingham, Alabama

**Keywords:** Demyelinating diseases, 4-Aminopyridine, Guillain-Barre syndrome, Rehabilitation, *List of abbreviations:* ECG, electrocardiogram, FDA, Food and Drug Administration, 4-AP, 4-aminopyridine, GBS, Guillain-Barré syndrome, MOS-12, Medical Outcomes Study 12-Item, MS, multiple sclerosis, PANAS, Positive and Negative Affect Schedule, VAS, visual analog scale

## Abstract

**Objective:**

To determine the safety and efficacy of orally delivered 4-aminopyridine (4-AP) in persons with Guillain-Barré Syndrome (GBS) >6 months from initial diagnosis.

**Design:**

A randomized, double-blind, placebo-controlled, crossover study.

**Setting:**

Tertiary care clinical outpatient program.

**Participants:**

Nineteen participants enrolled (14 male, 5 female; N=19), neurologic impairment secondary to GBS and functional loss on the FIM motor score (stable for ≥12mo) and >3.0 but <5.0 on the American Spinal Injury motor scale. Twelve participants (mean age, 59y; range, 23-77y) completed the study.

**Interventions:**

A 4-AP dose-escalation study with 8 weeks in each period with a 3-week washout period, followed by 3 months open-label follow-up.

**Main Outcome Measures:**

FIM motor score was the primary outcome measure; also evaluated were the American Spinal Injury motor strength score (all limbs), handheld dynamometer, 6-minute walk test, Medical Outcomes Study 12-Item Short Form, Center for Epidemiological Studies Depression scale, Positive and Negative Affect Schedule, pain, GBS disability scale, Jepsen-Taylor Hand Function Test, Minnesota Manual Dexterity Test and Minnesota Rate of Manipulation Test, Get Up and Go Test, McGill Pain Inventory, Craig Handicap Assessment and Reporting Technique, and participant self-evaluation.

**Results:**

Seven participants discontinued the study prematurely: 3 because of adverse events, 3 because of travel difficulties or relocation, and 1 because of pretreatment laboratory abnormalities. After removing 3 participants with maximum FIM scores, 4-AP arm trended superior to placebo (*P*=.065). Patients subjectively could always tell when they were on the active agent usually by tingling sensations or a sense of wellness. No statistically significant differences were found for other outcome measures although there were strong trends.

**Conclusions:**

This study demonstrates the safety of 4-AP in the patient population with GBS as the predominate goal of the study. A trend toward improved function after treatment was noted with most patients electing to stay on the medication after the trial.

## Highlights

• The demyelination of peripheral nerves due to Guillain-Barré syndrome can cause conduction blocks, similar to that seen in multiple sclerosis.
• 4-Aminopyridine conjugates can aid in reducing conduction blocks and may improve function in patients with demyelinating peripheral neuropathy.
• This study demonstrated the safety and the potential to improve the functional status in patients Guillain-Barré syndrome with chronic functional deficits.

Guillain-Barré syndrome (GBS) is an immunopathy associated with an acute, often fulminate, evolution of a demyelinating inflammatory polyradiculoneuropathy.^1^ It can affect anyone, regardless of age, sex, or ethnicity. GBS, also known as acute inflammatory demyelinating polyneuropathy, is the most common cause of acute nontraumatic neuromuscular paralysis in developed countries, afflicting approximately 0.4-1.9 cases/100,000 people annually but varying geographically with some areas up to 3.93 cases/100,000.^2^, ^3^, ^4^ Recent data indicate that up to 80% of patients with GBS have lifelong residual motor complaints, fatigue, and weakness and/or chronic pain.^5^^,^^6^

Early treatment with plasma exchange or intravenous immunoglobulin may lessen the severity of GBS and speed up the recovery process.^7^ However, there is no approved treatment of any kind for the debilitating fatigue and motor weakness in patients who have not fully recovered from GBS.^8^

4-aminopyridine (4-AP) is a specific blocker of voltage-dependent, fast-activating neuronal potassium channels.^9^, ^10^, ^11^, ^12^ 4-AP restores the action potential conduction in damaged, poorly myelinated nerve fibers by increasing the duration and amplitude of the compound action potential. It also directly enhances synaptic transmission at the synaptic bulb, thereby restoring conduction in those nerves with a conduction block.^13^, ^14^, ^15^ The mechanism of action is by blocking membrane potassium channels, thereby enhancing the voltage action potential that can extend the effect beyond damaged nodes of Ranvier from segmental demyelination.^16^, ^17^, ^18^

4-AP may also improve neuromuscular conduction at the level of the muscle by increasing transmitter release, thereby improving strength similar to the effect that aminopyridines in general have demonstrated in Lambert-Eaton syndrome.^13^ Both the restoration of the conduction block and improved synaptic transmission are mechanisms of action that may benefit the functional status in the patient population with chronic GBS.^13^, ^14^, ^15^, ^16^, ^17^, ^18^

4-AP is reported to be effective in patients with demyelination of the central nervous system, such as multiple sclerosis (MS) or spinal cord injury.^11^^,^^16^^,^^17^^,^^19^^,^^20^ Double-blind trials with 4-AP have demonstrated that patients with MS benefit from long-term administration of 4-AP, particularly regarding motor function and endurance.^11^^,^^17^ Clinical studies have demonstrated that 4-AP is associated with decreased pain and/or improved motor function.^11^^,^^19^^,^^20^ In 1 randomized, double-blind, placebo-controlled, crossover study involving 70 patients with MS, 4-AP administered for 12 weeks (maximum dose 0.5 mg/kg) was associated with significant improvements in the Kurtzke expanded disability status scale, activities of daily living, and several neurophysiological parameters.^12^ In a study involving 31 patients with MS followed for 6-32 months, ambulation and fatigue improved in 13 patients and visual function improved in 5 patients.^11^ Preliminary findings indicate that 4-AP is also associated with decreased pain in patients with MS.^11^^,^^19^^,^^20^, ^21^, ^22^

4-AP has been investigated in the treatment of motor weakness and fatigue in GBS and inflammatory polyneuropathy with preliminary findings that were very encouraging.^23^ After discussions with the Food and Drug Administration (FDA) it was decided another phase II trial should be performed with an emphasis on prescribed safety variables and specified coprimary physiological, functional outcome measures, and suggested secondary measures. Consequently, a follow-up phase IIB, randomized, double-blind, placebo-controlled, crossover, dose-escalation study was conducted to assess the safety and efficacy of 4-AP in participants with chronic motor and sensory deficits from GBS. This initial study emphasizes safety and investigation of potential efficacy outcome measures.

## Methods

This phase IIB, randomized, double-blind, placebo-controlled, crossover, dose-escalation study of the safety and efficacy of 4-AP in participants with GBS was similar to the phase IIA study,^23^ with the following exceptions: (1) double-blind treatment periods extended to 8 weeks; (2) placebo washout period increased to 3 weeks between treatment sequences; (3) inclusion criteria refined; and (4) additional exploratory outcome variables included.

Patients were randomized via a coin toss to determine which intervention was first. The research pharmacy kept all medication logs, and the research pharmacy issued all study medication. The study was performed over 3 years. All providers and coordinators were blinded as to which arm each patient was assigned. Institutional Review Board approval was obtained from the University of Alabama at Birmingham Human Investigation Committee.

### Inclusion criteria

Participants were required to meet all of the following criteria: (1) were male or female participants, aged 19-75 years, irrespective of race; (2) were able to and had voluntarily given informed consent before any study-specific procedures; (3) had a neurologic impairment secondary to GBS per National Institutes of Health clinical guidelines,^1^, ^2^, ^3^, ^4^ which had been stable for at least 12 months; (4) had a motor score that averaged between <5.0 but was >3.0 on the American Spinal Injury motor scale for all extremities; (5) were able and willing to comply with the protocol; and (6) and agreed to not change in their outpatient therapy or home exercise programs during enrollment in the study.

### Exclusion criteria

Participants were excluded from the study for any of the following: (1) they were pregnant or lactating or a woman of child-bearing potential not using reliable birth control or not surgically sterilized; (2) they had a history of seizures; (3) there was any evidence of upper motor neuron involvement; (4) there was any medical condition, including psychiatric disease, which would interfere with the interpretation of the results; (5) they had a known allergy to pyridine-containing substances; (6) their concomitant medications were at a stable dose/regimen <3 weeks, and/or the concomitant medications were expected to change during the study; (7) they had any history of drug or alcohol abuse within the past year; (8) they received an investigational drug within 30 days before the screening visit; (9) they had taken 4-AP in the past; (10) there was a problematic preexisting pain syndrome before the onset of GBS; (11) they had any history of cardiac abnormality or cardiac arrhythmia; and (12) they used any muscle relaxant or drugs with muscle-relaxant properties or drugs with alpha_2_-adrenergic receptor–blocking properties.

### Treatments

The study was carried out in a university-based outpatient physical medicine and rehabilitation clinic. This was a double-blind crossover study assigned via a coin toss to a treatment sequence (table 1). Placebo capsules were identical in appearance to the 4-AP^a,b,c^ (5.0 mg) capsules. Double-blind medication for each participant was code-labeled and individually packaged. The initial dose was 1 (5.0 mg) capsule/d at bedtime. The dosage was increased slowly with a target dose of 10 mg 3 times per day after 2 weeks of treatment, with continued treatment at 30 mg/d for an additional 6 weeks (8wk total). Participants underwent a 3-week placebo washout period before they were crossed over to the second treatment (8wk total).

**Table 1:** Treatment schedule

Period 1				Period 2		
Days*	Sequence A 4-AP	Sequence B		Days*	Sequence A	Sequence B 4-AP
1-2	5 mg/d	Placebo	Placebo Washout Days 61-82	83-84	Placebo	5 mg/d
3-4	5 mg 2 × /d	Placebo		85-86	Placebo	5 mg 2 × /d
5-6	5 mg 3 × /d	Placebo		87-88	Placebo	5 mg 3 × /d
7-8	5-5-10 mg	Placebo		89-90	Placebo	5-5-10 mg
9-10	5-10-10 mg	Placebo		91-92	Placebo	5-10-10 mg
11-60	10 mg 3 × /d	Placebo		93-142	Placebo	10 mg 3 × /d

NOTE. Dose escalation only applied when participants did not exhibit a dose-limiting toxicity defined as a drug-related adverse event severe enough to interfere with the participants’ daily activity. In the event participants experienced such toxicity, they were instructed to reduce the dose to the next lowest dosage level. If dose-limiting toxicity continued to occur, the participants were to be discontinued from the study. The maximum allowable dose of 4 AP was 30 mg/d

⁎ On clinic visit days, participants were to take their morning dose before coming to the clinic.

### Investigational medication

Information related to the chemistry and manufacture of tablets, including controls as well as the analysis of 4-AP supplied, are provided in the supplemental appendix S1 (available online only at http://www.archives-pmr.org/).^a-c^ The target dose of 4-AP^24^^,a,b,c^ in this study (30 mg 3 times per day) was based on review of safety and pharmacokinetic data in healthy adults and patients with MS or spinal cord injury.^7^^,^^8^ Dosages of up to a total of 30 mg/d have generally been well tolerated in these populations.^11^^,^^12^^,^^21^^,^^22^ 4-AP has a half-life of about 3.6 hours and is predominantly excreted by the kidney without biotransformation. Oral administration of 4-AP in doses significantly above 30 mg/d resulted in plasma levels in excess of 100 ng/mL.^21^^,^^24^

### Efficacy variables

Many of the efficacy variables were chosen after consultation with the United States FDA for the Investigational New Drug permit. The Neurologic Division of the Center for Drug Evaluation and Research of the FDA required the study to have coprimary neurologic variables covering both physiological and functional neurologic outcomes.

### Primary physiologic outcome measure: motor strength score

The American Spinal Injury motor score measures the strength of key muscles bilaterally in the upper and lower extremities. It is closely related to the American Spinal Injury Association motor scoring technique that is used throughout the rehabilitation community.^25^ This motor score scale grades motor strength of selected muscle groups on an integer scale ranging from 0 (paralysis) to 5 (normal).

### Primary functional outcome measure: FIM motor

FIM motor (13 items) is a widely used measure of functional independence in rehabilitation and has excellent validity and reliability.^26^^,^^27^ The FIM score ranges from 1 (total assistance) to 7 (complete independence).

### Secondary outcome measures

#### GBS disability scale

This scale assesses the extent of patient disability using an integer scale ranging from 0 (healthy) to 6 (dead).^7^

#### Hand-held dynamometer (grip strength)

The Jamar hand-held dynamometer is a reproducible method for evaluating maximal grip strength.^28^ Participants were evaluated 3 times in each hand with a calibrated Jamar dynamometerd with the highest grip strength recorded.

#### Jebsen-Taylor Hand Function Test

The Jebsen-Taylor Hand Function Test assesses functional endurance of the hands. It is an evaluation method that uses 7 tasks representative of everyday functional activities including handwriting.^29^

#### Minnesota Rate of Manipulation Test and the Minnesota Manual Dexterity Test

The Minnesota Rate of Manipulation Test and Minnesota Manual Dexterity Test involve repetitive movements of the hands using both the extrinsic and intrinsic muscles of the hands.^30^ Both of these tests use the same equipment and are easy to administer.

#### Get Up and Go Test

The Get Up and Go Test is a measurement of functional mobility and is also useful in following clinical change over time.^31^ This test measured how long it took participants to get up from a standard chair and walk 10 m with turning around. Participants were allowed to use their ambulatory assistive devices for this test.

#### 6-minute walk test

The 6-minute walk test is widely used to measure endurance in patients with functional limitations. It is well accepted by patients, is easily implemented, and has good reproducibility.^32^^,^^33^ The testing procedure followed the American Thoracic Society guidelines.^33^

#### Pain evaluations: McGill Pain Inventory and visual analog scale

Participants completed the McGill Pain Inventory questionnaire^34^^,^^35^ and the visual analog scale (VAS)^36^ for both intensity and unpleasant dimensions. The VAS measured pain at the time of the interview using a scale ranging from 1 (no pain) to 10 (worst). The McGill Pain Inventory also measured pain at the time of the interview, and as part of the McGill Pain Inventory, participants diagramed their pain locations. Where separate and distinct pain locations were identified, McGill Pain Inventory and VAS measures were taken for each location separately.

#### Depression questionnaires

Participants completed the Center for Epidemiologic Studies Depression Scale, which is a 20-item scale that was used as a covariate in analyses.^37^^,^^38^ Because 4-AP reportedly improves mood, its effect on the Positive and Negative Affect Schedule (PANAS) was also measured.^39^

#### Medical Outcomes Study 12-Item Short Form

The Medical Outcomes Study 12-Item (MOS-12) is a measurement of persons’ perception of their health status.^40^ It is highly correlated with the Medical Outcomes Study 36-Item Short Form.^41^ The MOS-12 provides 2 summary subscale scores: physical health and the mental health summary score

#### Participant self-evaluation

Participants recorded whether they had noticed changes in the following areas: bladder, bowel, sexual function, motor function, sensation, energy/stamina, or other. They also rated their satisfaction about the effects of the study medication.

### Safety variables

Safety was evaluated by examination of adverse events and changes in clinical laboratory values, vital signs, electrocardiograms (ECGs), and nerve conduction studies for changes in conduction velocity.

#### Adverse events

An adverse event was any undesirable event that occurred in a participant during the course of the study (or a reasonable time after study termination), whether or not that event was considered study drug related. Adverse effects associated with oral administration of 4-AP have included mild dizziness, lightheadedness, paresthesia/dysesthesia, nausea, and mild agitation.^10^^,^^20^^,^^22^^,^^23^^,^^42^ Doses >30 mg per day have induced confusional states (disorientation, agitation, anxiety), respiratory distress (dyspnea, hyperventilation), locomotor and balance problems, and epileptiform seizures.^21^^,^^23^^,^^24^^,^^42^ These plasma levels were also associated with seizure activity in some patients with MS.^10^^,^^20^^,^^21^^,^^23^^,^^24^^,^^42^ Cardiac abnormalities have been reported only with extremely high doses, beyond what is now recommended for treatment.^42^

#### Clinical laboratory tests

Clinical laboratory evaluations included blood chemistry and hematology tests and are listed in tables 5 and 6. Women of childbearing potential were given an additional urine pregnancy test during the study. The laboratory evaluations included chemistries of alanine aminotransferase, albumin, alkaline phosphatase, aspartate aminotransferase, bicarbonate, blood urea nitrogen, calcium, chloride, creatinine, lactate dehydrogenase, phosphorus, potassium, sodium, and total protein uric acid. The hematology evaluations included hematocrit, hemoglobin, platelet count, red blood cell count, red blood cell indices, white blood cell count, white blood cell differential, neutrophil count (absolute and %), lymphocyte count (absolute and %), monocyte count (absolute and %), eosinophil count (absolute and %), and basophil count (absolute and %).

#### Vital signs

Heart rate and blood pressure were taken at each clinic visit after the participant had been in a reclining position for 3 minutes. If the participant reported symptoms consistent with postural hypotension, additional blood pressure measures were taken and recorded in the participant's data worksheet.

#### Electrocardiograms

A standard 12-lead ECG was recorded at screening and at weeks 2, 4, and 8 of the first treatment period and at weeks 13, 15, and 19 of the second treatment period to assess for any changes, particularly ST segment duration changes and conduction abnormalities.

#### Nerve conduction studies

All participants had 2 upper motor (median and ulnar), 2 upper sensory (median and ulnar), 2 lower motor (tibial and peroneal), and 2 lower sensory (peroneal and sural) nerve conductions performed at screening and at week 8 in both arms.^43^^,^^44^ In addition, 1 upper and 1 lower F-wave were performed, which assesses for objective improvement in nerve conduction velocity with use of 4-AP.^45^ Nerve conduction velocities and amplitudes were performed for the median and peroneal nerves. Particular attention was given to the nerve conduction velocity, the terminal latency, changes in the negative to positive peak duration, and area under the curve. These are accepted parameters for evaluating demyelination using electrodiagnosis.

### Statistical and analytical plans

The efficacy analysis was performed on randomized participants who completed both treatment periods. The safety analysis was performed on all participants who received at least 1 dose of study medication (18 participants).

All data were reported as mean ± 1 SE and ± 1 SD. The Wilcoxon signed-rank test was used to test the significance of observed differences between baseline and after 8 weeks of continuous treatment for ordinally measured data (eg, GBS disability scale, motor strength, VAS). We assessed changes over time using Friedman's analysis, which is the nonparametric equivalent of the repeated measures analysis of variance model. Two-tailed tests were used, with *P*<.05 considered significant. Although nonparametric tests were used, data are presented as means and SDs to facilitate the interpretation of the magnitude and clinical significance of the results. The physiological motor score was obtained by combining both the upper extremity and lower extremity scores, which were then averaged for motor score. We similarly analyzed the average scores for the Jebsen-Taylor Hand Function Test, 6-minute walk test, GBS disability scale, pain (VAS, McGill, PANAS), Minnesota Manual Dexterity Test, MOS-12, and FIM motor.

All clinical laboratory tests, nerve conduction studies, and grip strength tests were assessed using a 2-tailed Student *t* test and/or a 1-way analysis of variance model with repeated measures.

## Results

Fourteen male and 5 female participants were in the study. The mean age of the study population was 59 years (range, 23-77y). Seven participants discontinued the study prematurely. Three discontinued because of adverse events that were considered likely related to 4-AP: 1 because of tremor, cramping, weakness, dizziness, ataxia, and diabetic hypoglycemia; 1 because of weakness, shaking (tremors), and postural hypotension; and 1 because of dizziness. Two participants discontinued because of travel difficulties and 1 because of relocation. One enrolled participant discontinued because of pretreatment laboratory abnormalities. The remaining 12 participants completed both periods of the crossover study.

### Primary Outcomes

#### Physiologic: motor scores

**Fig 1:**
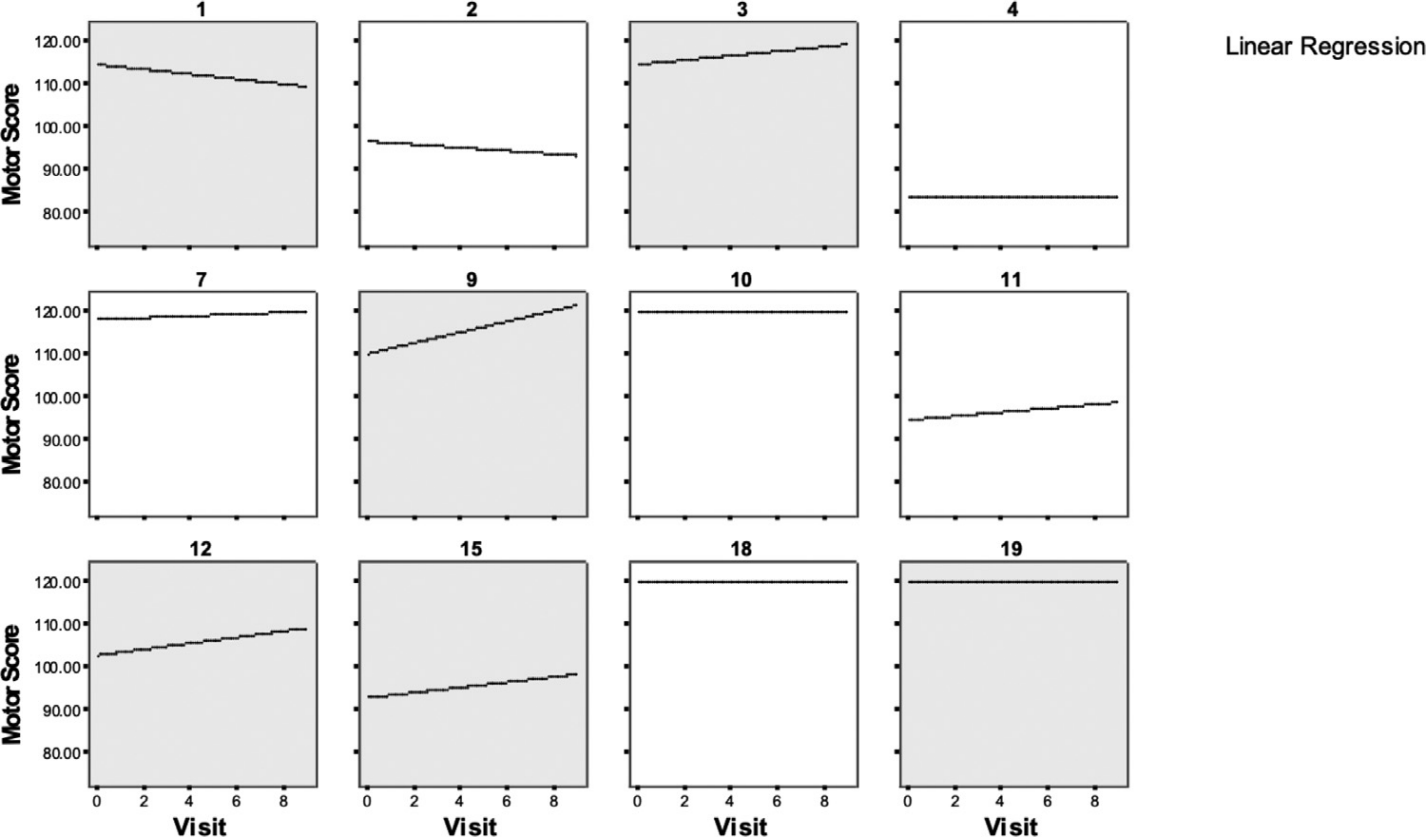
Motor strength scores by individual participant during 4-AP treatment. The gray highlighted plots indicate participants who received 4-AP in the first treatment sequence of the study.

The mean ± SD baseline motor strength score (all extremities combined) for study participants was 105.08±13.64. The maximum obtainable score on this measure is 120, with higher scores indicating greater muscle strength. Higher mean ± SE motor strength scores were found for placebo 109.6±2.2 compared with 4-AP treatment 107.2±2.2 (table 2). However, a trend toward statistical significance was apparent between 4-AP and placebo (F_1,10_=3.30, *P*=.10), although it was not in the desired direction. A ceiling effect on the motor strength scale was apparent for some participants but does not appear clinically meaningful (fig 1).

**Table 2:** Motor strength score: type 3 tests of fixed effects and means

Effect	Degrees of Freedom		F Value	*P* Value		
	Numerator	Denominator				
Treatment	1	9	3.30	.1026		

Effect	Treatment	Mean	SE	*df*	*t* Value	Pr>|*t*|
Treatment	4-AP	107.18	2.2437	9	47.77	<0.0001
Treatment	Placebo	109.65	2.2437	9	48.87	<0.0001

NOTE. Motor score strength scores and statistical evaluation using Wilcoxon signed-rank.

Abbrevation: Pr, *P* reference value.

#### Function: FIM motor scores

The mean ± SD baseline FIM motor score for all study participants was 83.58±10.80. The maximum score on the FIM motor scale is 91, with higher scores indicating greater functional independence. Table 3 presents the tests for the fixed effects in the model. There was an improvement in the motor scores with 4-AP treatment associated with higher FIM scores than with placebo treatment, indicating greater functional independence, but this fell short of statistical significance (*P*=.1205) (see table 3A). At the end of 8 weeks, the mean FIM score for 4-AP was 85.7 compared with 83.7 for placebo (see table 3B). The group on average, however, tended to have FIM scores near the upper limit at baseline, which produced a “ceiling effect,” that is, reduced the amount of change that can be detected with the FIM. Treatment effects were thus more difficult to detect because of the presence of ceiling effects.

**Table 3:** FIM motor scores and statistical evaluation using Wilcoxon signed-rank.

Table 3A FIM motor: type 3 tests of fixed effects						
Effect	Degrees of Freedom		F Value	*P* Value		
	Numerator	Denominator				
Treatment	1	10	2.88	.1205		
FIM motor: means						

Effect	Treatment	Estimate	SE	*df*	*t* Value	Pr>|*t*|
Treatment	4-AP	85.6667	0.7985	10	107.29	<0.0001
Treatment	Placebo	83.7500	0.7985	10	104.89	<0.0001

Table 3B FIM motor reanalysis: type 3 tests of fixed effects						
Effect	Degrees of Freedom		F Value	*P* Value		
	Numerator	Denominator				
Treatment	1	9	2.48	.1497		
FIM motor reanalysis: means						

Effect	Treatment	Estimate	SE	*df*	*t* Value	Pr>|*t*|
Treatment	4-AP	86.76	0.8534	9	101.66	<0.0001
Treatment	Placebo	84.86	0.8534	9	99.43	<0.0001

NOTE. FIM motor score scores and statistical evaluation using Wilcoxon signed-rank.

Abbrevation: Pr, *P* reference value.

**Fig 2:**
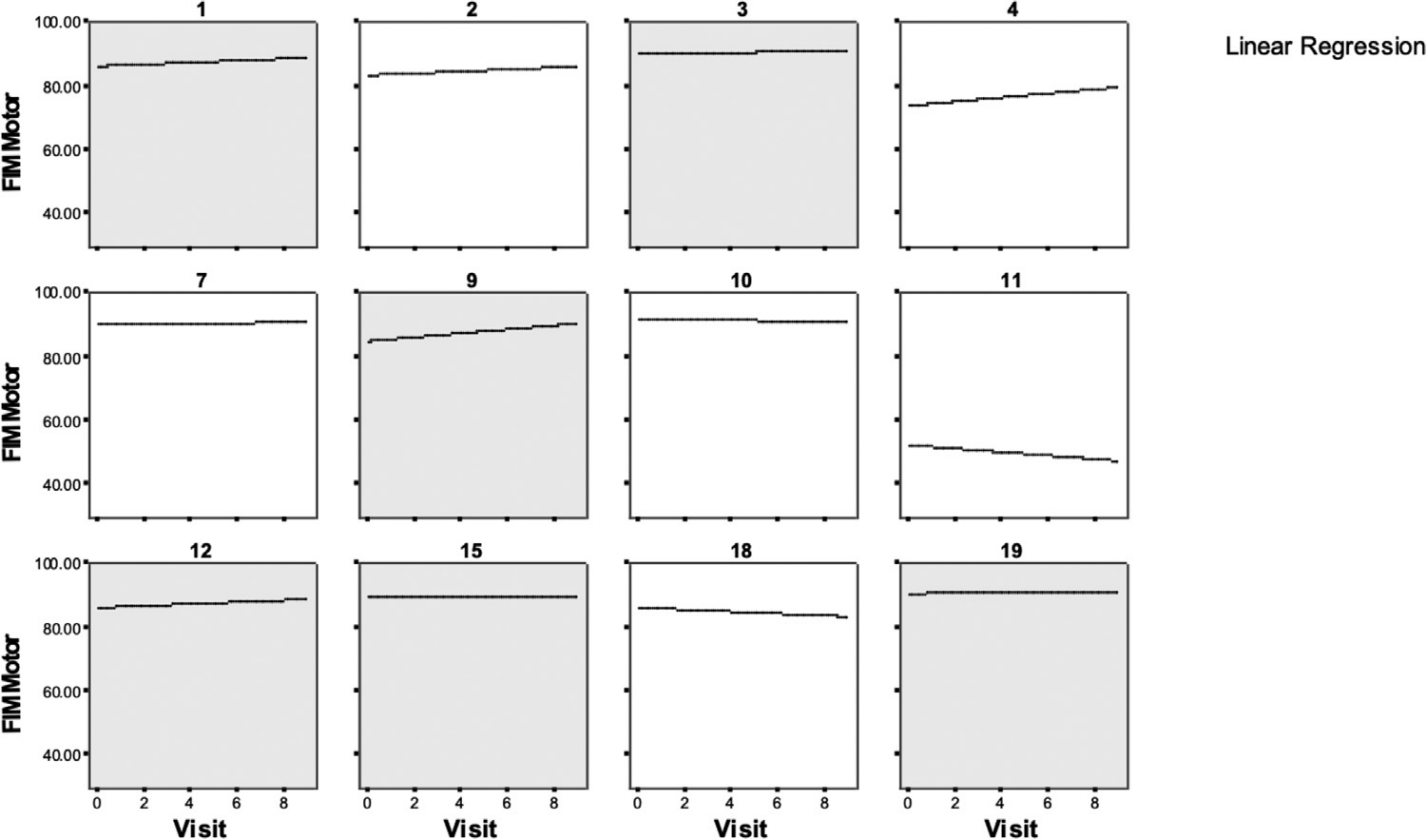
FIM motor scores by individual participant during 4-AP treatment. The highlighted plots indicate participants who received 4-AP in the first treatment sequence of the study.

FIM motor data were then examined by individual participant during 4-AP treatment (see table 3B) (fig 2). Fitted ordinary least squares trajectories were superimposed on the empirical response plots for participants in the following series of plots. A ceiling effect was clearly observed in the majority of the participants (see fig 2).

Visual inspection of the case report forms and original electronic database revealed some inconsistencies in FIM scores that could not be reconciled between the 2 sources of information for 1 participant (no. 4). FIM data for this participant were thus removed and the data reanalyzed (see table 3B). This revised analysis still failed to establish a clear improvement in motor function because there was a ceiling effect for a majority of the participants. This did not improve the results (*P*=.1497), although a positive trend was noted.

### Secondary outcomes

#### Functional

No statistically significant differences were found between 4-AP and placebo after 8 weeks of treatment for motor strength scores, bilateral grip strength (hand-held dynamometer), 6-minute walk test, MOS-12 physical component summary, Center for Epidemiologic Studies Depression Scale, and PANAS. Overall, there was a good deal of variability in the data, especially in the second treatment sequence (table 4).

**Table 4:** Changes from baseline of the efficacy parameter determined in the phase IIB trial

Parameters	Baseline	4-AP Wk 8	Placebo Wk 8	*P* Value
Grip strength (right hand)	49.4 lb (SE=39.0)	52.5 lb (SE=3.2)	52.4 lb (SE=3.2)	.97
Grip strength (left hand)	52.6 lb (SE=34.5)	52.3 lb (SE=3.4)	53.0 lb (SE=3.4)	.83
6-minute walk test	972 ft (SD=418)	1105 ft (SE=58)	1105 ft (SD=58)	.99
MOS-12	30.0 (SD=9.1)	34.7 (SD=2.2)	36.3 (SD=2.1)	.59
CES-D	9.4 (SD=9.8)	7.3 (SD=1.7)	5.2 (SD=1.7)	.42
PANAS (positive affect)	34.0 (SD=5.6)	36.0 (SE=2.9)	33.9 (SE=2.9)	.56
PANAS (negative affect)	16.8 (SD=5.6)	12.5 (SE=1.4)	10.8 (SE=1.4)	.29
Pain (VAS)	4.2 (SD=3.4)	3.0 (SD=1.2)	3.1 (SD=1.2)	.92

NOTE. Data presented as mean (SE/SD).

Abbreviation: CES-D, Center for Epidemiologic Studies Depression Scale.

#### Safety: laboratory and other tests

The 1 laboratory test that changed while on 4-AP was mean alkaline phosphatase, which increased statistically (*P*=.04), but this was not felt to be clinically important in any 1 patient treated and may be related to enzyme induction. Otherwise there were no other abnormal laboratory values discovered in individual participants, and the average values demonstrated no statistical trends or clinically significant changes throughout the study (tables 5 and 6). Urinalysis tests revealed no changes.

**Table 5:** Chemistry: paired change in serum samples test during 4-AP treatment (change from baseline to wk 8)

Parameters	Paired Differences				*t* Value	*df*	*P* Value*
			95% CI				
	Mean	SD	Lower	Upper			
Sodium (mEq/L)	−0.17	1.946	−1.40	1.07	−0.297	11	.772
Potassium (mEq/L)	0.000	0.4805	−0.305	0.305	0.000	11	>.99
Chloride (mEq/L)	0.58	2.109	−0.76	1.92	0.958	11	.359
Calcium (mEq/L)	0.175	0.2864	−0.007	0.357	2.116	11	.058
Bicarbonate (mEq/L)	−0.58	2.503	−2.17	1.01	−0.807	11	.437
Total protein (mg/dL)	0.110	0.4748	−0.230	0.450	0.733	9	.482
Glucose (mg/dL)	5.09	11.987	−2.96	13.14	1.409	10	.189
BUN (mg/dL)	2.33	4.271	−0.38	5.05	1.892	11	.085
Creatinine (mg/dL)	−0.017	0.1467	−0.110	0.077	−0.394	11	.701
Uric acid (mg/dL)	0.200	0.7274	−0.262	0.662	0.952	11	.361
Albumin (g/dL)	0.100	0.1581	−0.022	0.222	1.897	8	.094
Phosphorus (mg/dL)	0.282	0.4468	−0.018	0.582	2.092	10	.063
Total bilirubin (mg/dL)	0.010	0.2998	−0.204	0.224	0.105	9	.918
LDH (U/L)	23.41	65.899	−18.46	65.28	1.230	11	.244
AST (U/L)	1.60	7.834	−4.00	7.20	0.646	9	.535
ALT (U/L)	0.60	7.877	−5.03	6.23	0.241	9	.815
ALP (U/L)	3.50	4.673	0.16	6.84	2.369	9	.042

Abbreviations: ALP, alkaline phosphatase; ALT, alanine aminotransferase; AST, aspartate aminotransferase; BUN, blood urea nitrogen; CI, confidence interval; LDH, lactate dehydrogenase.

⁎ Two-tailed paired Student *t* test.

**Table 6:** Hematology: paired samples test during 4-AP treatment (change from baseline to week 8)

Parameters	Paired Differences				*t* Value	*df*	*P* Value
			95% CI				
	Mean	SD	Lower	Upper			*
PCV (%)	0.64	1.286	−0.23	1.50	1.641	10	.132
Hemoglobin (g/dL)	0.136	0.6217	−0.281	0.554	0.727	10	.484
RBC count (× 10^6^/µ/L)	0.0709	0.13736	−0.0214	0.1632	1.712	10	.118
Platelet count (× 10^3^/µ/L)	−9.236	72.4828	−57.931	39.458	−0.423	10	.682
WBC count (× 10^3^/µ/L)	−0.1991	1.38885	−1.1321	0.7340	−0.475	10	.645
WBC differential							
Segments (%)	−0.45	5.646	−4.25	3.34	−0.267	10	.795
Lymphocytes (%)	0.18	3.219	−1.98	2.34	0.187	10	.855
Monocytes (%)	0.55	2.505	−1.14	2.23	0.722	10	.487
Eosinophils (%)	−0.20	1.317	−1.14	0.74	−0.480	9	.642
Basophils (%)	0.20	0.632	−0.25	0.65	1.000	9	.343

Abbreviations: CI, confidence interval; PCV, packed cell volume; RBC, red blood cell; WBC, white blood cell.

⁎ Two-tailed paired Student *t* test.

No seizures were reported, nor were there any other clinically significant changes in laboratory tests, vital signs, ECG parameters, or nerve conduction studies in either amplitude or velocity of conduction.

## Discussion

The primary focus of this phase IIB trial was safety as required by the FDA. In this regard the study demonstrated excellent safety at the prescribed dosing and in the method of reaching the maximal dose. In this second phase IIB trial a trend for improvement of the FIM motor score was observed in this population with GBS, but the results are clearly inconclusive and not clinically meaningful for motor function as the primary outcome. A trend for improvement was observed for some endpoints. There were some indications of a carryover effect of 4-AP, indicating a prolonged neurologic effect long after the drug is cleared from the general systemic circulation of the body, but the design of the study and the small number of participants precludes any conclusions in this area. Those outcome measures that have more of a motor endurance measure appear to demonstrate more meaningful trends.

Similar to the findings with MS, there could be a distinct subgroup of patients with GBS who may benefit from the use of 4-AP for functional skills, either because of timing after illness or because of a specific functional deficit. The effect appears to be more activity based than a general improvement in motor power because the physiological motor scores did not markedly improve vs placebo. This study, along with a previous small study indicate that 4-AP needs further study in specific subgroups of demyelinating diseases.^23^

### Study limitations

**Fig 3:**
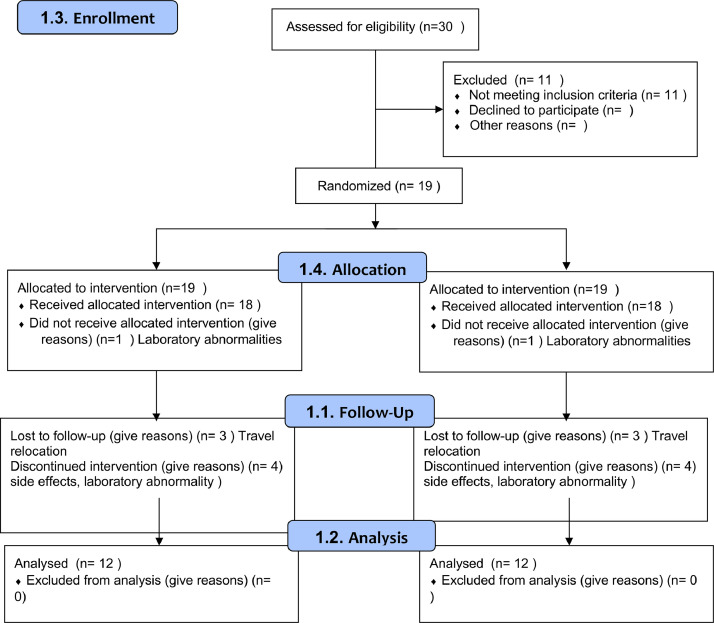
CONSORT flow diagram 4-AP in GBS. CONSORT, Consolidated Standards of Reporting Trials.

This preliminary study combined with a previous small study are limited in numbers because of the parameters of this orphan drug study by the US FDA after discussions. It is likely that because of the small sample size significance was not reached. This is supported by the fact that the variability of data were considerably high because of the diversity of the patient population. Patients with various levels of disability were recruited into the studies. The study's primary focus was on safety as required by the FDA. Patients could generally tell when they were on the drug. Three patients had to drop out, including 1 because of the changes in sensation, so adverse effects will need to be monitored closely if this goes to a phase III trial, which was recommended by the FDA in the next trial. It does provide some good data regarding the relevant outcome measures the FDA would like explored as well as data that could be used for a larger power analyses for a definitive study (fig 3).

## Conclusions

This trial along with a previously reported small trial are the first trials to have been performed to investigate the effects of 4-AP in the therapy of GBS including a control group (see fig 3).^23^ This study is inconclusive on the use of 4-AP in patients with chronic functional limitations from GBS several months from the acute occurrence.

There were indications of benefits of 4-AP in GBS because positive trends were also observed for other endpoints in the study. The patient comments indicated they had benefits and additional improvements, but more work would be necessary to define a subpopulation that may benefit, as there was in MS.^46^

Limited adverse effects occurred in our study, mostly associated with mild tremors that abated. Despite the previous report on dysaesthesias,^47^^,^^48^ this was not a reported problem for this study. However, all the patients felt they could tell when they were on the drug because of tingling and were noted to be all correct after unblinding.

There is significant interest in using this compounded medication in demyelinating diseases.^49^, ^50^, ^51^, ^52^ Clearly, this needs to be more carefully studied in humans before any conclusions may be drawn. This study does outline the primary outcome measures and has enough statistical input to estimate power analyses for selected outcome measures for a phase III Investigational New Drug trial.

## Suppliers

a. API; Regis Technologies Inc.
b. Study medication 5.0 mg 4-AP capsules and matching placebo including inactive ingredients of lactose and coloring); Scott Wepfer, RPh, The Compounding Shoppe.
c. Analysis of 4-AP purity and concentration; Analytical Research Labs.
d. Jamar dynamometer; Patterson Medical.

## Appendix Supplementary materials

Image, application 1
