# Supplementary material for: Phase IIB Randomized Trial on the Use of 4-Aminopyridine in Guillain-Barré Syndrome
Source: Arch Rehabil Res Clin Transl. 2021 Apr 8;3(2):100123. doi: 10.1016/j.arrct.2021.100123 (PMC8212006; doi:10.1016/j.arrct.2021.100123)
Supplement: Supplementary file 1 [file mmc1.docx]

| **Appendix 16.2.3.1. FIM™ Motor** | | | |
| --- | --- | --- | --- |
| **Subject**  **Number** | **Treatment** | **Study Visit** | **FIM™ Motor** |
| 1 | None | Baseline | 86 |
|  | 4-AP | Week 2 | 86 |
|  |  | Week 4 | 86 |
|  |  | Week 6 | 89 |
|  |  | Week 8 | 88 |
|  | Washout | Week 11 | 89 |
|  | Placebo | Week 13 | 89 |
|  |  | Week 15 | 87 |
|  |  | Week 17 | 81 87§ |
|  |  | Week 19 | 90 |
| 2 | None | Baseline | 84 |
|  | Placebo | Week 2 | 84 |
|  |  | Week 4 | 89 |
|  |  | Week 6 | 83 |
|  |  | Week 8 | 83 82§ |
|  | Washout | Week 11 | 83 |
|  | 4-AP | Week 13 | 77 83§ |
|  |  | Week 15 | 84 |
|  |  | Week 17 | 87 |
|  |  | Week 19 | 90 |
| 3 | None | Baseline | 90 |
|  | 4-AP | Week 2 | 91 |
|  |  | Week 4 | 90 |
|  |  | Week 6 | 91 |
|  |  | Week 8 | 91 |
|  | Washout | Week 11 | 90 |
|  | Placebo | Week 13 | 91 |
|  |  | Week 15 | 91 |
|  |  | Week 17 | 91 |
|  |  | Week 19 | 91 |
| 4‡ | None | Baseline | 69 69§ |
|  | Placebo | Week 2 | 76 76§ |
|  |  | Week 4 | 82 75§ |
|  |  | Week 6 | 75 75§ |
|  |  | Week 8 | 70 80§ |
|  | Washout | Week 11 | 74 82§ |
|  | 4-AP | Week 13 | 70 77§ |
|  |  | Week 15 | 84 84§ |
|  |  | Week 17 | 70 76§ |
|  |  | Week 19 | 75 75§ |
| 5* | None | Baseline | 91 |
|  | Placebo | Week 2 | 91 |
|  |  | Week 4 | 91 |
|  |  | Week 6 | 91 |
|  |  | Week 8 | 91 |
|  | Washout | Week 11 | 91 |
|  | 4-AP | Week 13 | 91 |
|  |  | Week 15 |  |
|  |  | Week 17 |  |
|  |  | Week 19 |  |
| 6* | None | Baseline | 88 |
|  | 4-AP | Week 2 |  |
|  |  | Week 4 |  |
|  |  | Week 6 |  |
|  |  | Week 8 |  |
|  | Washout | Week 11 |  |
|  | Placebo | Week 13 |  |
|  |  | Week 15 |  |
|  |  | Week 17 |  |
|  |  | Week 19 |  |
| 7 | None | Baseline | 91 |
|  | Placebo | Week 2 | 90 |
|  |  | Week 4 | 91 |
|  |  | Week 6 | 90 |
|  |  | Week 8 | 91 |
|  | Washout | Week 11 | 91 |
|  | 4-AP | Week 13 | 91 |
|  |  | Week 15 | 91 |
|  |  | Week 17 | 90 |
|  |  | Week 19 | 91 |
| 8† | None | Baseline | 37 |
| 9 | None | Baseline | 88 |
|  | 4-AP | Week 2 | 86 |
|  |  | Week 4 | 86 88§ |
|  |  | Week 6 | 71 77§ |
|  |  | Week 8 | 88 |
|  | Washout | Week 11 | 90 |
|  | Placebo | Week 13 | 90 |
|  |  | Week 15 | 91 |
|  |  | Week 17 | 90 |
|  |  | Week 19 | 90 |
| 10 | None | Baseline | 90 |
|  | Placebo | Week 2 | 90 91§ |
|  |  | Week 4 | 98 91§ |
|  |  | Week 6 | 91 |
|  |  | Week 8 | 91 |
|  | Washout | Week 11 | 91 |
|  | 4-AP | Week 13 | 91 |
|  |  | Week 15 | 91 |
|  |  | Week 17 | NA |
|  |  | Week 19 | 91 |
| 11 | None | Baseline | 55 |
|  | Placebo | Week 2 | 55 |
|  |  | Week 4 | 49 55§ |
|  |  | Week 6 | NA |
|  |  | Week 8 | 47 |
|  | Washout | Week 11 | 32 |
|  | 4-AP | Week 13 | 58 |
|  |  | Week 15 | NA |
|  |  | Week 17 | 34 |
|  |  | Week 19 | 64 |
| 12 | None | Baseline | 86 |
|  | 4-AP | Week 2 | 86 |
|  |  | Week 4 | 88 |
|  |  | Week 6 | 87 |
|  |  | Week 8 | 86 |
|  | Washout | Week 11 | 89 |
|  | Placebo | Week 13 | 89 |
|  |  | Week 15 | 88 |
|  |  | Week 17 | 89 |
|  |  | Week 19 | 88 |
| 13* | None | Baseline | 87 |
|  | Placebo | Week 2 | 84 88§ |
|  |  | Week 4 | 86 |
|  |  | Week 6 | 89 |
|  |  | Week 8 | 85 |
|  | Washout | Week 11 | 86 |
|  | 4-AP | Week 13 | 84 80§ |
|  |  | Week 15 | 80 |
|  |  | Week 17 |  |
|  |  | Week 19 |  |
| 14* | None | Baseline | 82 |
|  | Placebo | Week 2 | 78 |
|  |  | Week 4 | 83 |
|  |  | Week 6 | 82 |
|  |  | Week 8 |  |
|  | Washout | Week 11 |  |
|  | 4-AP | Week 13 |  |
|  |  | Week 15 |  |
|  |  | Week 17 |  |
|  |  | Week 19 |  |
| 15 | None | Baseline | 89 |
|  | 4-AP | Week 2 | 87 |
|  |  | Week 4 | 96 88§ |
|  |  | Week 6 | 88 |
|  |  | Week 8 | 89 |
|  | Washout | Week 11 | 89 |
|  | Placebo | Week 13 | 90 |
|  |  | Week 15 | 89 |
|  |  | Week 17 | 90 |
|  |  | Week 19 | 89 |
| 16* | None | Baseline | 90 |
|  | 4-AP | Week 2 |  |
|  |  | Week 4 |  |
|  |  | Week 6 |  |
|  |  | Week 8 |  |
|  | Washout | Week 11 |  |
|  | Placebo | Week 13 |  |
|  |  | Week 15 |  |
|  |  | Week 17 |  |
|  |  | Week 19 |  |
| 17* | None | Baseline | 87 |
|  | Placebo | Week 2 | 84 |
|  |  | Week 4 | 90 |
|  |  | Week 6 |  |
|  |  | Week 8 |  |
|  | Washout | Week 11 |  |
|  | 4-AP | Week 13 |  |
|  |  | Week 15 |  |
|  |  | Week 17 |  |
|  |  | Week 19 |  |
| 18 | None | Baseline | 84 |
|  | Placebo | Week 2 | 86 89§ |
|  |  | Week 4 | 86 |
|  |  | Week 6 | 86 |
|  |  | Week 8 | 84 |
|  | Washout | Week 11 | 84 |
|  | 4-AP | Week 13 | 84 |
|  |  | Week 15 | 84 |
|  |  | Week 17 | 84 |
|  |  | Week 19 | 84 |
| 19 | None | Baseline | 91 |
|  | 4-AP | Week 2 | 90 |
|  |  | Week 4 | 91 |
|  |  | Week 6 | 91 |
|  |  | Week 8 | 91 |
|  | Washout | Week 11 | 91 |
|  | Placebo | Week 13 | 91 |
|  |  | Week 15 | 91 |
|  |  | Week 17 | 91 |
|  |  | Week 19 | 91 |
| **Abbreviations:** 4-AP = 4-aminopyridine; NA = not available  **Note:** The FIM™ motor (13 items) ranges from 1 to 7, with 1 (total assistance) being the worst possible score and 7 (complete independence) being the best possible score. The result presented is the total score for the 13 items assessed (maximum score = 91).  * Subject prematurely discontinued from the study.  † Subject was withdrawn before receipt of study medication because of pretreatment laboratory abnormalities.  ‡ Subject was not included in the reanalysis of FIM™ data because of some inconsistencies in FIM™ scores, which could not be reconciled, between the case report form and the electronic database.  § Value used in the analysis based on review of case report forms. This represents a worst case scenario, where potential discrepancies in the data were found. | | | |

| **Appendix 16.2.3.2. Motor Strength** | | | |
| --- | --- | --- | --- |
| **Subject Number** | **Treatment** | **Study Visit** | **Motor Strength Score (Upper/Lower Extremities)** |
| 1 | None | Baseline | 110 |
|  | 4-AP | Week 2 | 118 |
|  |  | Week 4 | 107 |
|  |  | Week 6 | 116 |
|  |  | Week 8 | 116 |
|  | Washout | Week 11 | 116 |
|  | Placebo | Week 13 | 116 |
|  |  | Week 15 | 88 108‡ |
|  |  | Week 17 | 100 |
|  |  | Week 19 | 113 |
| 2 | None | Baseline | 96 |
|  | Placebo | Week 2 | 96 |
|  |  | Week 4 | 96 |
|  |  | Week 6 | 96 |
|  |  | Week 8 | 96 |
|  | Washout | Week 11 | 92 |
|  | 4-AP | Week 13 | 96 |
|  |  | Week 15 | 99 |
|  |  | Week 17 | 96 |
|  |  | Week 19 | 88 |
| 3 | None | Baseline | 111 |
|  | 4-AP | Week 2 | 115 |
|  |  | Week 4 | 117 |
|  |  | Week 6 | 120 |
|  |  | Week 8 | 116 |
|  | Washout | Week 11 | 118 |
|  | Placebo | Week 13 | 120 |
|  |  | Week 15 | 114 |
|  |  | Week 17 | 120 |
|  |  | Week 19 | 118 |
| 4 | None | Baseline | 84 |
|  | Placebo | Week 2 | 84 |
|  |  | Week 4 | 74 |
|  |  | Week 6 | 90 |
|  |  | Week 8 | 83 |
|  | Washout | Week 11 | 84 |
|  | 4-AP | Week 13 | 88 |
|  |  | Week 15 | 88 |
|  |  | Week 17 | 88 |
|  |  | Week 19 | 74 |
|  | Open-label, 4-AP | Month 1 | 74 |
|  |  | Month 2 | 116 108‡ |
|  |  | Month 3 | 88 |
| 5* | None | Baseline | 118 |
|  | Placebo | Week 2 | 116 |
|  |  | Week 4 | 116 |
|  |  | Week 6 | 114 |
|  |  | Week 8 | 120 |
|  | Washout | Week 11 | 120 |
|  | 4-AP | Week 13 | 120 |
|  |  | Week 15 | 120 |
|  |  | Week 17 |  |
|  |  | Week 19 |  |
| 6* | None | Baseline | 96 |
|  | 4-AP | Week 2 |  |
|  |  | Week 4 |  |
|  |  | Week 6 |  |
|  |  | Week 8 |  |
|  | Washout | Week 11 |  |
|  | Placebo | Week 13 |  |
|  |  | Week 15 |  |
|  |  | Week 17 |  |
|  |  | Week 19 |  |
| 7 | None | Baseline | 116 |
|  | Placebo | Week 2 | 119 |
|  |  | Week 4 | 120 |
|  |  | Week 6 | 119 |
|  |  | Week 8 | 120 |
|  | Washout | Week 11 | 118 |
|  | 4-AP | Week 13 | 120 |
|  |  | Week 15 | 118 |
|  |  | Week 17 | 120 |
|  |  | Week 19 | 120 |
|  | Open-label, 4-AP | Month 1 | 120 |
|  |  | Month 2 | 120 |
|  |  | Month 3 | 118 |
| 8† | None | Baseline | 96 |
| 9 | None | Baseline | 103 111‡ |
|  | 4-AP | Week 2 | 94 104‡ |
|  |  | Week 4 | 118 |
|  |  | Week 6 | 118 |
|  |  | Week 8 | 120 |
|  | Washout | Week 11 | 118 |
|  | Placebo | Week 13 | 118 |
|  |  | Week 15 | 120 |
|  |  | Week 17 | 118 |
|  |  | Week 19 | 120 |
| 10 | None | Baseline | 120 |
|  | Placebo | Week 2 | 120 |
|  |  | Week 4 | 120 |
|  |  | Week 6 | 120 |
|  |  | Week 8 | 120 |
|  | Washout | Week 11 | 120 |
|  | 4-AP | Week 13 | 120 |
|  |  | Week 15 | 120 |
|  |  | Week 17 | NA |
|  |  | Week 19 | 120 |
|  | Open-label, 4-AP | Month 1 | 120 |
|  |  | Month 2 | 120 |
|  |  | Month 3 | 120 |
| 11 | None | Baseline | 80 86‡ |
|  | Placebo | Week 2 | 102 |
|  |  | Week 4 | 96 |
|  |  | Week 6 | 94 |
|  |  | Week 8 | 102 |
|  | Washout | Week 11 | 102 |
|  | 4-AP | Week 13 | 102 96‡ |
|  |  | Week 15 | NA |
|  |  | Week 17 | 90 |
|  |  | Week 19 | 102 |
|  | Open-label, 4-AP | Month 1 | 102 100‡ |
|  |  | Month 2 | 96 |
|  |  | Month 3 | 97 |
| 12 | None | Baseline | 95 |
|  | 4-AP | Week 2 | 108 |
|  |  | Week 4 | 105 103‡ |
|  |  | Week 6 | 118 113‡ |
|  |  | Week 8 | 101 |
|  | Washout | Week 11 | 113 |
|  | Placebo | Week 13 | 105 |
|  |  | Week 15 | 107 |
|  |  | Week 17 | 108 |
|  |  | Week 19 | 107 |
|  | Open-label, 4-AP | Month 1 | 106 |
|  |  | Month 2 | 114 |
|  |  | Month 3 | NA 115‡ |
| 13* | None | Baseline | 118 |
|  | Placebo | Week 2 | 110 |
|  |  | Week 4 | 110 |
|  |  | Week 6 | 120 |
|  |  | Week 8 | 120 |
|  | Washout | Week 11 | 120 |
|  | 4-AP | Week 13 | 116 100‡ |
|  |  | Week 15 | 100 116‡ |
|  |  | Week 17 |  |
|  |  | Week 19 |  |
| 14* | None | Baseline | 90 |
|  | Placebo | Week 2 | 95 |
|  |  | Week 4 | 98 |
|  |  | Week 6 | 107 |
|  |  | Week 8 |  |
|  | Washout | Week 11 |  |
|  | 4-AP | Week 13 |  |
|  |  | Week 15 |  |
|  |  | Week 17 |  |
|  |  | Week 19 |  |
| 15 | None | Baseline | 92 |
|  | 4-AP | Week 2 | 92 |
|  |  | Week 4 | 92 |
|  |  | Week 6 | 108 |
|  |  | Week 8 | 90 |
|  | Washout | Week 11 | 90 94‡ |
|  | Placebo | Week 13 | 98 |
|  |  | Week 15 | 97 |
|  |  | Week 17 | 102 |
|  |  | Week 19 | 96 |
|  | Open-label, 4-AP | Month 1 | 112 |
|  |  | Month 2 | 92 |
|  |  | Month 3 | 92 |
| 16* | None | Baseline | 106 |
|  | 4-AP | Week 2 |  |
|  |  | Week 4 |  |
|  |  | Week 6 |  |
|  |  | Week 8 |  |
|  | Washout | Week 11 |  |
|  | Placebo | Week 13 |  |
|  |  | Week 15 |  |
|  |  | Week 17 |  |
|  |  | Week 19 |  |
| 17* | None | Baseline | 112 |
|  | Placebo | Week 2 | 112 |
|  |  | Week 4 | 105 |
|  |  | Week 6 |  |
|  |  | Week 8 |  |
|  | Washout | Week 11 |  |
|  | 4-AP | Week 13 |  |
|  |  | Week 15 |  |
|  |  | Week 17 |  |
|  |  | Week 19 |  |
| 18 | None | Baseline | 120 |
|  | Placebo | Week 2 | 120 |
|  |  | Week 4 | 120 |
|  |  | Week 6 | 120 |
|  |  | Week 8 | 120 |
|  | Washout | Week 11 | 120 |
|  | 4-AP | Week 13 | 120 |
|  |  | Week 15 | 120 |
|  |  | Week 17 | 120 |
|  |  | Week 19 | 120 |
| 19 | None | Baseline | 120 |
|  | 4-AP | Week 2 | 120 |
|  |  | Week 4 | 120 |
|  |  | Week 6 | 120 |
|  |  | Week 8 | 120 |
|  | Washout | Week 11 | 120 |
|  | Placebo | Week 13 | 120 |
|  |  | Week 15 | 120 |
|  |  | Week 17 | 120 |
|  |  | Week 19 | 120 |
| **Abbreviations:** 4-AP = 4-aminopyridine; NA = not available  **Note:** Motor score grades motor strength of selected muscle groups on a scale ranging from 0 (absent, total paralysis) to 5 (normal, active movement against full resistance). The result presented is the total score for upper and lower limb motor movements.  * Subject prematurely discontinued from the study.  † Subject was withdrawn before receipt of study medication because of pretreatment laboratory abnormalities.  ‡ Value used in the analysis based on review of case report forms. | | | |

| **Appendix 16.2.3.3. Grip Strength (Dynamometer)** | | | | | | |
| --- | --- | --- | --- | --- | --- | --- |
| **Subject**  **Number** | **Treatment** | **Study Visit** | **Right Hand** | | **Left Hand** | |
|  |  |  | **Trial 1** | **Trial 2** | **Trial 1** | **Trial 2** |
| 1 | None | Baseline | 55 | 53 | 63 | 60 |
|  | 4-AP | Week 2 | 54 | 51 | 65 | 64 67‡ |
|  |  | Week 4 | 46 | 39 | 55 | 48 |
|  |  | Week 6 | 57 | 42 | 66 | 62 |
|  |  | Week 8 | 46 | 34 | 53 | 53 |
|  | Washout | Week 11 | 60 | 60 | 60 | 63 |
|  | Placebo | Week 13 | 60 | 60 57‡ | 60 73‡ | 63 73‡ |
|  |  | Week 15 | 42 | 40 | 57 | 54 |
|  |  | Week 17 | 60 | 56 | 71 | 62 |
|  |  | Week 19 | 60 | 56 | 71 | 66 |
| 2 | None | Baseline | 27 | 30 | 20 | 20 |
|  | Placebo | Week 2 | 10 | 9 | 7 | 9 |
|  |  | Week 4 | 18 | 18 | 16 | 12 |
|  |  | Week 6 | 1 | 2 | 2 | 4 |
|  |  | Week 8 | 4 | 4 | 0 | 0 |
|  | Washout | Week 11 | 30 | 22 | 15 | 16 |
|  | 4-AP | Week 13 | 18 | 16 | 18 | 9 |
|  |  | Week 15 | 24 | 23 | 16 | 15 |
|  |  | Week 17 | 18 | 21 | 20 | 30 |
|  |  | Week 19 | 30 | 25 | 23 | 25 |
| 3 | None | Baseline | 50 | 52 | 40 | 41 |
|  | 4-AP | Week 2 | 64 | 59 | 52 | 51 |
|  |  | Week 4 | 53 | 55 | 56 | 46 |
|  |  | Week 6 | 51 | 54 | 64 | 60 |
|  |  | Week 8 | 65 | 75 | 64 | 54 |
|  | Washout | Week 11 | 80 | 72 | 69 | 60 |
|  | Placebo | Week 13 | 64 | 59 | 55 | 64 |
|  |  | Week 15 | 63 | 65 | 71 | 64 |
|  |  | Week 17 | 72 | 80 | 67 | 65 |
|  |  | Week 19 | 75 | 71 | 68 | 61 |
| 4 | None | Baseline | 0 | 0 | 0 | 0 |
|  | Placebo | Week 2 | 14 | 13 | 7 | 6 |
|  |  | Week 4 | 1 | 1 | 0 | 0 |
|  |  | Week 6 | 2 | 0 | 0 | 0 |
|  |  | Week 8 | 16 | 16 | 8 | 6 |
|  | Washout | Week 11 | 0.5 | 9 | 0 | 0 |
|  | 4-AP | Week 13 | 13 | 13 | 8 | 6 |
|  |  | Week 15 | 14 | 14 | 7 | 7 |
|  |  | Week 17 | 13 | 14 | 9 | 6 |
|  |  | Week 19 | 15 | 20 | 8 | 9 |
| 5* | None | Baseline | 45 | 73 | 73 | 70 |
|  | Placebo | Week 2 | 20 | 24 | 41 | 48 |
|  |  | Week 4 | 34 | 38 | 60 | 60 |
|  |  | Week 6 | 65 | 75 | 74 | 80 |
|  |  | Week 8 | 45 | 58 | 67 | 75 |
|  | Washout | Week 11 | 70 | 74 | 84 | 90 |
|  | 4-AP | Week 13 | 70 | 74 | 84 | 90 |
|  |  | Week 15 |  |  |  |  |
|  |  | Week 17 |  |  |  |  |
|  |  | Week 19 |  |  |  |  |
| 6* | None | Baseline | 40 | 44 | 40 | 41 |
|  | 4-AP | Week 2 |  |  |  |  |
|  |  | Week 4 |  |  |  |  |
|  |  | Week 6 |  |  |  |  |
|  |  | Week 8 |  |  |  |  |
|  | Washout | Week 11 |  |  |  |  |
|  | Placebo | Week 13 |  |  |  |  |
|  |  | Week 15 |  |  |  |  |
|  |  | Week 17 |  |  |  |  |
|  |  | Week 19 |  |  |  |  |
| 7 | None | Baseline | 107 | 120 | 110 | 110 |
|  | Placebo | Week 2 | 120 | 122 | 100 | 115 |
|  |  | Week 4 | 130 | 130 | 125 | 125 |
|  |  | Week 6 | 130 | 125 | 120 | 120 |
|  |  | Week 8 | 125 | 118 | 115 | 116 |
|  | Washout | Week 11 | 140 | 153 | 150 | 130 |
|  | 4-AP | Week 13 | 96 | 134 | 108 | 102 |
|  |  | Week 15 | 120 | 118 | 115 | 110 |
|  |  | Week 17 | 128 | 131 | 119 | 111 |
|  |  | Week 19 | 108 | 118 | 93 | 91 |
| 8† | None | Baseline | 45 | 52 | 47 | 54 |
| 9 | None | Baseline | 0 | 0 | 80 | 85 |
|  | 4-AP | Week 2 | 0 | 0 | 80 | 80 |
|  |  | Week 4 | 5 | 2 | 68 | 85 |
|  |  | Week 6 | 2 | 2 | 75 | 72 |
|  |  | Week 8 | 2 | 2 | 80 | 77 |
|  | Washout | Week 11 | 2 | 1 | 69 | 74 |
|  | Placebo | Week 13 | 1 | 1 | 73 | 72 |
|  |  | Week 15 | 3 | 0 | 60 | 75 |
|  |  | Week 17 | 5 | 1 | 73 | 70 |
|  |  | Week 19 | 6 | 1 | 72 | 71 |
| 10 | None | Baseline | 102 | 100 | 95 | 90 |
|  | Placebo | Week 2 | 104 | 95 | 110 | 100 |
|  |  | Week 4 | 111 | 100 | 87 | 100 |
|  |  | Week 6 | 113 | 112 | 112 | 115 |
|  |  | Week 8 | 114 | 107 | 105 | 105 |
|  | Washout | Week 11 | 110 | 103 | 115 | 113 |
|  | 4-AP | Week 13 | 120 | 110 | 115 | 105 |
|  |  | Week 15 | 110 | 108 | 106 | 100 |
|  |  | Week 17 | NA | NA | NA | NA |
|  |  | Week 19 | 116 | 108 | 107 | 104 |
| 11 | None | Baseline | 23 | 18 | 26 | 22 |
|  | Placebo | Week 2 | 22 | 20 | 21 | 18 |
|  |  | Week 4 | 29 | 20 | 27 | 19 |
|  |  | Week 6 | NA | NA | NA | NA |
|  |  | Week 8 | 23 | 22 | 24 | 25 |
|  | Washout | Week 11 | 22 | 24 | 21 | 25 |
|  | 4-AP | Week 13 | 24 | 26 | 23 | 25 |
|  |  | Week 15 | NA | NA | NA | NA |
|  |  | Week 17 | 26 | 23 | 23 | 25 |
|  |  | Week 19 | 26 | 24 | 23 | 20 |
| 12 | None | Baseline | 42 | 35 | 41 | 40 |
|  | 4-AP | Week 2 | 35 | 30 | 32 | 34 |
|  |  | Week 4 | 35 | 33 | 31 | 30 |
|  |  | Week 6 | 33 | 34 | 34 | 34 |
|  |  | Week 8 | 34 | 39 | 34 | 33 |
|  | Washout | Week 11 | 40 | 38 | 35 | 33 |
|  | Placebo | Week 13 | 40 | 37 | 37 | 35 |
|  |  | Week 15 | 40 | 40 | 35 | 35 |
|  |  | Week 17 | 39 | 35 | 35 | 32 |
|  |  | Week 19 | 40 | 38 | 36 | 36 |
| 13* | None | Baseline | 32 | 40 | 30 | 34 |
|  | Placebo | Week 2 | 45 | 43 | 45 | 38 |
|  |  | Week 4 | 44 | 46 | 35 | 42 |
|  |  | Week 6 | 45 | 40 | 32 | 35 |
|  |  | Week 8 | 41 | 43 | 39 | 36 |
|  | Washout | Week 11 | 43 | 35 | 35 | 30 |
|  | 4-AP | Week 13 | 39 35‡ | 40 45‡ | 28 30‡ | 34 35‡ |
|  |  | Week 15 | 35 | 45 | 30 | 35 |
|  |  | Week 17 |  |  |  |  |
|  |  | Week 19 |  |  |  |  |
| 14* | None | Baseline | 30 | 29 | 35 | 35 |
|  | Placebo | Week 2 | 30 | 31 | 36 | 37 |
|  |  | Week 4 | 29 | 30 | 34 | 35 |
|  |  | Week 6 | 26 | 30 | 34 | 34 |
|  |  | Week 8 |  |  |  |  |
|  | Washout | Week 11 |  |  |  |  |
|  | 4-AP | Week 13 |  |  |  |  |
|  |  | Week 15 |  |  |  |  |
|  |  | Week 17 |  |  |  |  |
|  |  | Week 19 |  |  |  |  |
| 15 | None | Baseline | 18 | 17 | 15 | 15 |
|  | 4-AP | Week 2 | 8 | 10 | 4 | 3 |
|  |  | Week 4 | 20 | 19 | 16 | 15 |
|  |  | Week 6 | 19 | 12 | 8 | 6 |
|  |  | Week 8 | 15 | 16 | 10 | 14 |
|  | Washout | Week 11 | 14 | 16 | 11 | 11 |
|  | Placebo | Week 13 | 19 | 20 | 15 | 15 |
|  |  | Week 15 | 8 | 9 | 4 | 5 |
|  |  | Week 17 | 15 | 16 | 9 | 11 |
|  |  | Week 19 | 16 | 15 | 7 | 6 |
| 16* | None | Baseline | 80 | 76 | 85 | 84 |
|  | 4-AP | Week 2 |  |  |  |  |
|  |  | Week 4 |  |  |  |  |
|  |  | Week 6 |  |  |  |  |
|  |  | Week 8 |  |  |  |  |
|  | Washout | Week 11 |  |  |  |  |
|  | Placebo | Week 13 |  |  |  |  |
|  |  | Week 15 |  |  |  |  |
|  |  | Week 17 |  |  |  |  |
|  |  | Week 19 |  |  |  |  |
| 17* | None | Baseline | 85 | 90 | 80 | 90 |
|  | Placebo | Week 2 | 90 | 98 | 100 | 100 |
|  |  | Week 4 | 95 | 94 | 80 | 86 |
|  |  | Week 6 |  |  |  |  |
|  |  | Week 8 |  |  |  |  |
|  | Washout | Week 11 |  |  |  |  |
|  | 4-AP | Week 13 |  |  |  |  |
|  |  | Week 15 |  |  |  |  |
|  |  | Week 17 |  |  |  |  |
|  |  | Week 19 |  |  |  |  |
| 18 | None | Baseline | 90 | 100 | 75 | 80 |
|  | Placebo | Week 2 | 89 | 84 | 78 | 71 |
|  |  | Week 4 | 96 | 90 | 80 | 70 |
|  |  | Week 6 | 95 | 95 | 80 | 70 |
|  |  | Week 8 | 96 | 85 | 80 | 65 |
|  | Washout | Week 11 | 90 | 100 | 85 | 80 |
|  | 4-AP | Week 13 | 90 | 93 | 70 | 70 |
|  |  | Week 15 | 82 | 87 | 74 | 75 |
|  |  | Week 17 | 100 | 92 | 85 | 79 |
|  |  | Week 19 | 97 | 93 | 80 | 78 |
| 19 | None | Baseline | 73 | 73 | 75 | 60 |
|  | 4-AP | Week 2 | 86 | 76 | 69 | 67 |
|  |  | Week 4 | 50 | 60 | 78 | 65 |
|  |  | Week 6 | 84 | 72 | 75 | 75 |
|  |  | Week 8 | 80 | 72 | 65 | 56 |
|  | Washout | Week 11 | 61 | 65 | 70 | 70 |
|  | Placebo | Week 13 | 81 | 65 | 72 | 67 |
|  |  | Week 15 | 81 | 79 | 67 | 65 |
|  |  | Week 17 | 72 | 73 | 70 | 70 |
|  |  | Week 19 | 80 | 70 | 65 | 65 |
|  | Open-label, 4-AP | Month 1 | NA | NA | NA | NA |
|  |  | Month 2 | 55 | 53 | 63 | 60 |
|  |  | Month 3 | 54 | 51 | 65 | 64 |
| **Abbreviations:** 4-AP = 4-aminopyridine; NA = not available  **Note:** A calibrated Jamar dynamometer was used to measure grip strength in each hand (pounds).  * Subject prematurely discontinued from the study.  † Subject was withdrawn before receipt of study medication because of pretreatment laboratory abnormalities.  ‡ Value used in the analysis of “maximum grip strength” based on review of case report forms. | | | | | | |

| **Appendix 16.2.3.4. 6-Minute Walk Test** | | | |
| --- | --- | --- | --- |
| **Subject**  **Number** | **Treatment** | **Study Visit** | **Distance Covered (feet)** |
| 1 | None | Baseline | 1272 |
|  | 4-AP | Week 2 | 1238 |
|  |  | Week 4 | 1227 |
|  |  | Week 6 | 1413 |
|  |  | Week 8 | 1239 |
|  | Washout | Week 11 | 1275 |
|  | Placebo | Week 13 | 1275 |
|  |  | Week 15 | 1319 |
|  |  | Week 17 | 1332 |
|  |  | Week 19 | 1306 |
| 2 | None | Baseline | 926 |
|  | Placebo | Week 2 | 936 |
|  |  | Week 4 | 980 |
|  |  | Week 6 | 870 |
|  |  | Week 8 | 857 |
|  | Washout | Week 11 | 851 |
|  | 4-AP | Week 13 | 1027 |
|  |  | Week 15 | 1020 |
|  |  | Week 17 | 1068 |
|  |  | Week 19 | 1056 |
| 3 | None | Baseline | 1275 |
|  | 4-AP | Week 2 | 1350 |
|  |  | Week 4 | 1223 |
|  |  | Week 6 | 1474 |
|  |  | Week 8 | 1425 |
|  | Washout | Week 11 | 1425 |
|  | Placebo | Week 13 | 1400 |
|  |  | Week 15 | 1386 |
|  |  | Week 17 | 1425 |
|  |  | Week 19 | 1509 |
| 4 | None | Baseline | 179 |
|  | Placebo | Week 2 | 195 |
|  |  | Week 4 | 216 |
|  |  | Week 6 | 288 |
|  |  | Week 8 | 321 |
|  | Washout | Week 11 | 303 |
|  | 4-AP | Week 13 | 338 |
|  |  | Week 15 | 402 |
|  |  | Week 17 | 404 |
|  |  | Week 19 | 325 |
| 5* | None | Baseline | 1326 |
|  | Placebo | Week 2 | 1246 |
|  |  | Week 4 | 1170 |
|  |  | Week 6 | 1241 |
|  |  | Week 8 | 1275 |
|  | Washout | Week 11 | 1276 |
|  | 4-AP | Week 13 | 1275 |
|  |  | Week 15 |  |
|  |  | Week 17 |  |
|  |  | Week 19 |  |
| 6* | None | Baseline | 1519 |
|  | 4-AP | Week 2 |  |
|  |  | Week 4 |  |
|  |  | Week 6 |  |
|  |  | Week 8 |  |
|  | Washout | Week 11 |  |
|  | Placebo | Week 13 |  |
|  |  | Week 15 |  |
|  |  | Week 17 |  |
|  |  | Week 19 |  |
| 7 | None | Baseline | 1320 |
|  | Placebo | Week 2 | 1425 |
|  |  | Week 4 | 1546 |
|  |  | Week 6 | 1529 |
|  |  | Week 8 | 1583 |
|  | Washout | Week 11 | 1575 |
|  | 4-AP | Week 13 | 1575 |
|  |  | Week 15 | 1650 |
|  |  | Week 17 | 1650 |
|  |  | Week 19 | 1677 |
| 8† | None | Baseline | 18 |
| 9 | None | Baseline | 1217 |
|  | 4-AP | Week 2 | 1171 |
|  |  | Week 4 | 1153 |
|  |  | Week 6 | 1150 |
|  |  | Week 8 | 1056 |
|  | Washout | Week 11 | 1116 |
|  | Placebo | Week 13 | 1125 |
|  |  | Week 15 | 1142 |
|  |  | Week 17 | 1125 |
|  |  | Week 19 | 1118 |
| 10 | None | Baseline | 1050 |
|  | Placebo | Week 2 | 1350 |
|  |  | Week 4 | 1425 |
|  |  | Week 6 | 1425 |
|  |  | Week 8 | 1425 |
|  | Washout | Week 11 | 1487 |
|  | 4-AP | Week 13 | 1500 |
|  |  | Week 15 | 1500 |
|  |  | Week 17 | NA |
|  |  | Week 19 | 1410 |
| 11 | None | Baseline | 200 |
|  | Placebo | Week 2 | 198 |
|  |  | Week 4 | 158 |
|  |  | Week 6 | NA |
|  |  | Week 8 | 160 |
|  | Washout | Week 11 | 178 |
|  | 4-AP | Week 13 | 157 |
|  |  | Week 15 | NA |
|  |  | Week 17 | 168 |
|  |  | Week 19 | 150 |
| 12 | None | Baseline | 789 |
|  | 4-AP | Week 2 | 785 |
|  |  | Week 4 | 830 |
|  |  | Week 6 | 780 |
|  |  | Week 8 | 825 |
|  | Washout | Week 11 | 825 |
|  | Placebo | Week 13 | 826 |
|  |  | Week 15 | 894 |
|  |  | Week 17 | 816 |
|  |  | Week 19 | 851 |
| 13* | None | Baseline | 859 |
|  | Placebo | Week 2 | 909 |
|  |  | Week 4 | 912 |
|  |  | Week 6 | 967 |
|  |  | Week 8 | 966 |
|  | Washout | Week 11 | 1012 |
|  | 4-AP | Week 13 | 825 962‡ |
|  |  | Week 15 | 962 |
|  |  | Week 17 |  |
|  |  | Week 19 |  |
| 14* | None | Baseline | 525 |
|  | Placebo | Week 2 | 483 |
|  |  | Week 4 | 575 |
|  |  | Week 6 | 596 |
|  |  | Week 8 |  |
|  | Washout | Week 11 |  |
|  | 4-AP | Week 13 |  |
|  |  | Week 15 |  |
|  |  | Week 17 |  |
|  |  | Week 19 |  |
| 15 | None | Baseline | 875 |
|  | 4-AP | Week 2 | 1226 |
|  |  | Week 4 | 1388 |
|  |  | Week 6 | 1401 |
|  |  | Week 8 | 1420 |
|  | Washout | Week 11 | 1350 |
|  | Placebo | Week 13 | 1348 |
|  |  | Week 15 | 1154 |
|  |  | Week 17 | 1217 |
|  |  | Week 19 | 1220 |
| 16* | None | Baseline | 1409 |
|  | 4-AP | Week 2 |  |
|  |  | Week 4 |  |
|  |  | Week 6 |  |
|  |  | Week 8 |  |
|  | Washout | Week 11 |  |
|  | Placebo | Week 13 |  |
|  |  | Week 15 |  |
|  |  | Week 17 |  |
|  |  | Week 19 |  |
| 17* | None | Baseline | 1228 |
|  | Placebo | Week 2 | 1344 |
|  |  | Week 4 | 1500 |
|  |  | Week 6 |  |
|  |  | Week 8 |  |
|  | Washout | Week 11 |  |
|  | 4-AP | Week 13 |  |
|  |  | Week 15 |  |
|  |  | Week 17 |  |
|  |  | Week 19 |  |
| 18 | None | Baseline | 1062 |
|  | Placebo | Week 2 | 900 |
|  |  | Week 4 | 1084 |
|  |  | Week 6 | 978 |
|  |  | Week 8 | 1050 |
|  | Washout | Week 11 | 1022 |
|  | 4-AP | Week 13 | 1108 |
|  |  | Week 15 | 900 |
|  |  | Week 17 | 1125 |
|  |  | Week 19 | 1064 |
| 19 | None | Baseline | 1496 |
|  | 4-AP | Week 2 | 1500 |
|  |  | Week 4 | 1650 |
|  |  | Week 6 | 1800 |
|  |  | Week 8 | 1610 |
|  | Washout | Week 11 | 1611 |
|  | Placebo | Week 13 | 1558 |
|  |  | Week 15 | 1589 |
|  |  | Week 17 | 1812 |
|  |  | Week 19 | 1855 |
|  | Open-label, 4-AP | Month 1 | NA |
|  |  | Month 2 | 1272 |
|  |  | Month 3 | 1238 |
| **Abbreviations:** 4-AP = 4-aminopyridine; NA = not available  **Note:** The 6-minute walk test measures the total distance (in feet) subjects are able to walk over a 6-minute period.  * Subject prematurely discontinued from the study.  † Subject was withdrawn before receipt of study medication because of pretreatment laboratory abnormalities.  ‡ Value reported is based on review of case report forms. | | | |

| **Appendix 16.2.3.8. Pain (Visual Analog Scale)** | | | |
| --- | --- | --- | --- |
| **Subject Number** | **Treatment** | **Study Visit** | **Visual Analog Scale‡** |
| 1 | None | Baseline | 4 |
|  | 4-AP | Week 2 | 0 |
|  |  | Week 4 | NA |
|  |  | Week 6 | 0 |
|  |  | Week 8 | 0 |
|  | Washout | Week 11 | 0 |
|  | Placebo | Week 13 | 0 |
|  |  | Week 15 | 0 |
|  |  | Week 17 | 0 |
|  |  | Week 19 | 0 |
| 2 | None | Baseline | NA |
|  | Placebo | Week 2 | NA |
|  |  | Week 4 | 5 |
|  |  | Week 6 | 0 |
|  |  | Week 8 | 0 |
|  | Washout | Week 11 | 0 |
|  | 4-AP | Week 13 | 0 |
|  |  | Week 15 | 0 |
|  |  | Week 17 | 0 |
|  |  | Week 19 | 0 |
| 3 | None | Baseline | 5 |
|  | 4-AP | Week 2 | 2 |
|  |  | Week 4 | 0 |
|  |  | Week 6 | 0 |
|  |  | Week 8 | NA |
|  | Washout | Week 11 | 0 |
|  | Placebo | Week 13 | 0 |
|  |  | Week 15 | 0 |
|  |  | Week 17 | 0 |
|  |  | Week 19 | 0 |
| 4 | None | Baseline | 3 |
|  | Placebo | Week 2 | 0 |
|  |  | Week 4 | 0 |
|  |  | Week 6 | 3 |
|  |  | Week 8 | NA |
|  | Washout | Week 11 | 0 |
|  | 4-AP | Week 13 | 0 |
|  |  | Week 15 | 0 |
|  |  | Week 17 | 0 |
|  |  | Week 19 | 0 |
| 5* | None | Baseline | NA |
|  | Placebo | Week 2 | NA |
|  |  | Week 4 | NA |
|  |  | Week 6 | NA |
|  |  | Week 8 | NA |
|  | Washout | Week 11 | NA |
|  | 4-AP | Week 13 | NA |
|  |  | Week 15 |  |
|  |  | Week 17 |  |
|  |  | Week 19 |  |
| 6* | None | Baseline | NA |
|  | 4-AP | Week 2 |  |
|  |  | Week 4 |  |
|  |  | Week 6 |  |
|  |  | Week 8 |  |
|  | Washout | Week 11 |  |
|  | Placebo | Week 13 |  |
|  |  | Week 15 |  |
|  |  | Week 17 |  |
|  |  | Week 19 |  |
| 7 | None | Baseline | 8 |
|  | Placebo | Week 2 | 8 |
|  |  | Week 4 | 0 |
|  |  | Week 6 | 0 |
|  |  | Week 8 | 0 |
|  | Washout | Week 11 | 0 |
|  | 4-AP | Week 13 | 0 |
|  |  | Week 15 | 5 |
|  |  | Week 17 | 5 |
|  |  | Week 19 | 6 |
|  | Open-label, 4-AP | Month 1 | 4 |
|  |  | Month 2 | 4 |
|  |  | Month 3 | 0 |
| 8† | None | Baseline | NA |
| 9 | None | Baseline | 6 |
|  | 4-AP | Week 2 | 5 |
|  |  | Week 4 | 5 |
|  |  | Week 6 | 5 |
|  |  | Week 8 | 5 |
|  | Washout | Week 11 | 5 |
|  | Placebo | Week 13 | 5 |
|  |  | Week 15 | 5 |
|  |  | Week 17 | 5 |
|  |  | Week 19 | 5 |
| 10 | None | Baseline | NA |
|  | Placebo | Week 2 | 10 |
|  |  | Week 4 | 8 |
|  |  | Week 6 | 0 |
|  |  | Week 8 | 9 |
|  | Washout | Week 11 | 8 |
|  | 4-AP | Week 13 | 7 |
|  |  | Week 15 | 8 |
|  |  | Week 17 | NA |
|  |  | Week 19 | 10 |
|  | Open-label, 4-AP | Month 1 | 8 |
|  |  | Month 2 | 8 |
|  |  | Month 3 | 8 |
| 11 | None | Baseline | 7 |
|  | Placebo | Week 2 | 8 |
|  |  | Week 4 | 8 |
|  |  | Week 6 | 7 |
|  |  | Week 8 | 10 |
|  | Washout | Week 11 | 8 |
|  | 4-AP | Week 13 | 8 |
|  |  | Week 15 | NA |
|  |  | Week 17 | 7 |
|  |  | Week 19 | 5 |
|  | Open-label, 4-AP | Month 1 | 8 |
|  |  | Month 2 | 0 |
|  |  | Month 3 | 0 |
| 12 | None | Baseline | 0 |
|  | 4-AP | Week 2 | 0 |
|  |  | Week 4 | 0 |
|  |  | Week 6 | 0 |
|  |  | Week 8 | 0 |
|  | Washout | Week 11 | 0 |
|  | Placebo | Week 13 | 0 |
|  |  | Week 15 | 0 |
|  |  | Week 17 | NA |
|  |  | Week 19 | 0 |
|  | Open-label, 4-AP | Month 1 | 0 |
|  |  | Month 2 | 0 |
|  |  | Month 3 | 0 |
| 13* | None | Baseline | 10 |
|  | Placebo | Week 2 | 10 |
|  |  | Week 4 | 10 |
|  |  | Week 6 | 10 |
|  |  | Week 8 | NA |
|  | Washout | Week 11 | 9 |
|  | 4-AP | Week 13 | 0 |
|  |  | Week 15 | 0 |
|  |  | Week 17 |  |
|  |  | Week 19 |  |
| 14* | None | Baseline | NA |
|  | Placebo | Week 2 | NA |
|  |  | Week 4 | NA |
|  |  | Week 6 | NA |
|  |  | Week 8 |  |
|  | Washout | Week 11 |  |
|  | 4-AP | Week 13 |  |
|  |  | Week 15 |  |
|  |  | Week 17 |  |
|  |  | Week 19 |  |
| 15 | None | Baseline | 9 |
|  | 4-AP | Week 2 | 9 |
|  |  | Week 4 | 10 |
|  |  | Week 6 | 8 |
|  |  | Week 8 | 10 |
|  | Washout | Week 11 | 6 |
|  | Placebo | Week 13 | 10 |
|  |  | Week 15 | 7 |
|  |  | Week 17 | 4 |
|  |  | Week 19 | 10 |
|  | Open-label, 4-AP | Month 1 | 10 |
|  |  | Month 2 | 9 |
|  |  | Month 3 | 8 |
| 16* | None | Baseline | NA |
|  | 4-AP | Week 2 |  |
|  |  | Week 4 |  |
|  |  | Week 6 |  |
|  |  | Week 8 |  |
|  | Washout | Week 11 |  |
|  | Placebo | Week 13 |  |
|  |  | Week 15 |  |
|  |  | Week 17 |  |
|  |  | Week 19 |  |
| 17* | None | Baseline | NA |
|  | Placebo | Week 2 | NA |
|  |  | Week 4 | NA |
|  |  | Week 6 |  |
|  |  | Week 8 |  |
|  | Washout | Week 11 |  |
|  | 4-AP | Week 13 |  |
|  |  | Week 15 |  |
|  |  | Week 17 |  |
|  |  | Week 19 |  |
| 18 | None | Baseline | 0 |
|  | Placebo | Week 2 | 0 |
|  |  | Week 4 | 0 |
|  |  | Week 6 | 0 |
|  |  | Week 8 | 0 |
|  | Washout | Week 11 | 0 |
|  | 4-AP | Week 13 | 0 |
|  |  | Week 15 | NA |
|  |  | Week 17 | 0 |
|  |  | Week 19 | 0 |
| 19 | None | Baseline | 0 |
|  | 4-AP | Week 2 | 3 |
|  |  | Week 4 | 0 |
|  |  | Week 6 | 0 |
|  |  | Week 8 | 0 |
|  | Washout | Week 11 | 0 |
|  | Placebo | Week 13 | 0 |
|  |  | Week 15 | 0 |
|  |  | Week 17 | 0 |
|  |  | Week 19 | 0 |
|  | Open-label, 4-AP | Month 1 | NA |
|  |  | Month 2 | NA |
|  |  | Month 3 | 7 |
| **Abbreviations:** 4-AP = 4-aminopyridine, NA = not available  **Note:** Visual analog scale ranges from 1 to 10: 1 = no pain and 10 = pain as bad as it could be.  * Subject prematurely discontinued from the study.  † Subject was withdrawn before receipt of study medication because of pretreatment laboratory abnormalities.  ‡ VAS data were obtained from the case report forms. | | | |

| **Appendix 16.2.6. Nerve Conduction Studies** | | | | | | | |
| --- | --- | --- | --- | --- | --- | --- | --- |
|  |  |  | **Motor Nerve Conduction‡** | | | | |
| **Subject Number** | **Treatment** | **Study Visit** | **Median** | **Ulnar Distal** | **Ulnar Proximal** | **Peroneal** | **Tibial** |
| 1 | None | Baseline | 23 | 60 | 73 | 42 | 42 |
|  | 4-AP | Week 8 | 18 | 64 | 77 | 45 | 40 |
|  | Placebo | Week 19 | 24 | 55 | 85 | 43 | 41 |
| 2 | None | Baseline | 50 | 57 | 57 | 39 | 39 |
|  | Placebo | Week 8 | 51 | 59 | 83 | 41 | 39 |
|  | 4-AP | Week 19 | 50 | 57 | 61 | 38 | 35 |
| 3 | None | Baseline | 52 | 64 | 58 | 53 | 51 |
|  | 4-AP | Week 11 | 55 | 64 | 57 | 51 | 52 |
|  | Placebo | Week 19 | 57 | 63 | 62 | 53 | 51 |
| 4 | None | Baseline | 42 | 59 | 71 | NA | NA |
|  | Placebo | Week 8 | 44 | 56 | 47 | NA | NA |
|  | 4-AP | Week 19 | 46 | 53 | 53 | NA | NA |
| 5* | None | Baseline | 53 | 55 | 42 | 51 | 50 |
|  | Placebo | Week 8 | 55 | 58 | 37 | 50 | 47 |
|  | 4-AP | Week 19 |  |  |  |  |  |
| 6* | None | Baseline | 42 | 45 | 38 | NA | NA |
|  | 4-AP | Week 8 |  |  |  |  |  |
|  | Placebo | Week 19 |  |  |  |  |  |
| 7 | None | Baseline | 54 | 56 | 50 | NA | NA |
|  | Placebo | Week 8 | 54 | 51 | 53 | NA | 33 |
|  | 4-AP | Week 19 | 53 | 53 | 57 | NA | 40 |
| 8† | None | Baseline | 36 | 40 | 54 | 29 | NA |
| 9 | None | Baseline | NA | 42 | 31 | 38 | 35 |
|  | 4-AP | Week 8 | NA | 41 | 35 | 38 | 44 |
|  | Placebo | Week 17 | NA | 41 | 38 | 36 | 38 |
| 10 | None | Baseline | 51 | 56 | 40 | 40 | 41 |
|  | Placebo | Week 8 | 48 | 55 | 51 | 29 | 43 |
|  | 4-AP | Week 19 | 48 | 54 | 44 | 32 | 43 |
| 11 | None | Baseline | 38 | 42 | 24 | NA | NA |
|  | Placebo | Week 8 | 33 | 35 | 27 | NA | NA |
|  | 4-AP | Week 19 | 38 | 31 | 33 | NA | NA |
| 12 | None | Baseline | 43 | 80 | 23 | NA | NA |
|  | 4-AP | Week 8 | 38 | 57 | 40 | NA | NA |
|  | Placebo | Week 19 | 50 | 52 | 53 | NA | NA |
| 13* | None | Baseline | 50 | 59 | 67 | 54 | 54 |
|  | Placebo | Week 8 | 46 | 61 | 54 | 52 | 52 |
|  | 4-AP | Week 19 |  |  |  |  |  |
| 14* | None | Baseline | 42 | NA | NA | NA | NA |
| 15 | None | Baseline | 46 | 46 | 54 | 42 | 33 |
|  | 4-AP | Week 8 | 31 | 49 | 58 | 39 | 29 |
|  | Placebo | Week 19 | 49 | 52 | 62 | 40 | 38 |
| 16* | None | Baseline | 51 | 63 | 77 | 30 | 30 |
| 17* | None | Baseline | 52 | 58 | 59 | 44 | 45 |
| 18 | None | Baseline | 55 | 59 | 50 | 48 | 51 |
|  | Placebo | Week 8 | 51 | 58 | 62 | 49 | 51 |
|  | 4-AP | Week 19 | 52 | 56 | 50 | 44 | 46 |
| 19 | None | Baseline | 38 | 45 | 39 | NA | 29 |
|  | 4-AP | Week 8 | 42 | 46 | 41 | 20 | 32 |
|  | Placebo | Week 19 | 42 | 47 | 44 | 28 | 8 |
| **Abbreviation:** 4-AP = 4-aminopyridine; NA = not available  * Subject prematurely discontinued from the study.  † Subject was withdrawn before receipt of study medication because of pretreatment laboratory abnormalities.  ‡ Nerve conduction data were obtained from the case report. | | | | | | | |
